# Supplementary material for: Hospitalization costs for COVID-19 in Ethiopia: Empirical data and analysis from Addis Ababa’s largest dedicated treatment center
Source: PLoS One. 2022 Jan 21;17(1):e0260930. doi: 10.1371/journal.pone.0260930 (PMC8782501; doi:10.1371/journal.pone.0260930)
Supplement: S1 Appendix — (DOCX) [file pone.0260930.s001.docx]

**Supplementary web appendix I**

1. **Table 1: Description of cases by COVID-19 disease severity**

| **Moderate disease** | **Pneumonia** | Adolescent or adult with clinical signs of pneumonia (fever, cough, dyspnea, fast breathing) but no signs of severe pneumonia, including SpO_2_ ≥ 90% on room air.  Child with clinical signs of non-severe pneumonia (cough or difficulty breathing + fast breathing and/or chest indrawing) and no sign of severe pneumonia.  Fast breathing (in breaths/min): < 2 months: ≥ 60; 2-11 months: ≥ 50; 1-5 years: ≥ 40. |
| --- | --- | --- |
| **Severe disease** | **Severe pneumonia** | Adolescent or adult with clinical signs of pneumonia (fever, cough, dyspnea, fast breathing) plus one of the following: respiratory rate > 30 breaths/min; severe respiratory distress; or SpO2 < 90% on room air.  Child with clinical signs of pneumonia (cough or difficulty breathing) + at least one of the following:   - Central cyanosis or SpO_2_ < 90%: severe respiratory distress (e.g. fast breathing, grunting, very severe chest indrawing); general danger sign: inability to breastfeed or drink, lethargy or unconsciousness, or convulsions. - Fast breathing (in breaths/min): < 2 months: ≥ 60; 2-11 months: ≥ 50; 1-5 years: ≥ 40. |
| **Critical disease** | **Acute respiratory distress syndrome (ARDS)** | Onset: within 1 week of a known clinical insult (i.e. pneumonia) or new or worsening respiratory symptoms.  Chest imaging: (radiograph, CT scan, or lung ultrasound): bilateral opacities, not fully explained by volume overload, lobar or lung collapse, or nodules.  Origin of pulmonary infiltrates: respiratory failure not fully explained by cardiac failure or fluid overload. Need objective assessment (e.g. echocardiography) to exclude hydrostatic cause of infiltrates/edema if no risk factor present.  Oxygenation impairment in adults:   - Mild ARDS: 200mmHg < PaO_2_/FiO_2_ ≤ 300mmHg (with PEEP of CPAP ≥ 5 cmH_2_O) - Moderate ARDS: 100mmHg < PaO_2_/FiO_2_ ≤ 200mmHg (with PEEP of CPAP ≥ 5 cmH_2_O) - Severe ARDS: PaO_2_/FiO_2_ ≤ 100mmHg (with PEEP of CPAP ≥ 5 cmH_2_O)   Oxygenation impairment in children [use oxygenation index (OI) or oxygen saturation index (OSI):   - Mild ARDS (invasively ventilated): 4 ≤ OI < 8 or 5 ≤ OSI < 7.5. - Moderate ARDS (invasively ventilated): 8 ≤ OI < 16 or 7.5 ≤ OSI < 12.3. - Severe ARDS (invasively ventilated): OI ≥ 16 or OSI ≥ 12.3. |
| **Critical disease** | **Sepsis**  **Septic shock** | Adults: acute life threatening organ dysfunction caused by a dysregulated host response to suspected or proven infection. Signs of organ dysfunction include: altered mental status, difficult or fast breathing, low oxygen saturation, reduced urine output, fast heart rate, weak pulse, cold extremities or low blood pressure, skin mottling, laboratory evidence of coagulopathy, thrombocytopenia, acidosis, high lactate, or hyperbilirubinemia.  Children: suspected or proven infection and ≥ 2 age-based systemic inflammatory response syndrome (SIRS) criteria of which one must be abnormal temperature or white blood cell count.  Adults: persistent hypotension despite volume resuscitation, requiring vasopressors to maintain mean arterial pressure ≥ 65 mmHg and serum lactate level > 2 mmol/L.  Children: any hypotension (SBP < 5^th^ centile or > 2 SD below normal for age) or two or three of the following: altered mental status; bradycardia or tachycardia [heart rate < 90 beats per minute (bpm) or > 160 bpm in infants and heart <70 bpm or > 150 bpm in children]; prolonged capillary refill (> 2 sec) or weak pulse; fast breathing; mottled or cool skin or petechial or purpuric rash; high lactate; reduced urine output; hyperthermia or hypothermia. |

1. **Case management protocol for Corona Virus Disease-19 (COVID-19) in Ethiopia**
2. **Management of mild pneumonia**

Empiric oral antibiotics when needed; Amoxicillin 500mg oral (PO) twice daily (BID) or Amoxicillin- clavulanate (Augmentin) 2g PO BID for 7-10 days + Azithromycin 500mg PO for 3 days

1. **Management of severe Pneumonia**

- Provide oxygen supplementation with a target of oxygen saturation (SpO_2_) ≥ 90% for adults and SpO_2_ > 92-94% for pregnant mothers and children.
- Conservative intravenous (IV) fluid management should be instituted.
- Empiric antimicrobials should be started after taking specimen for culture and sensitivity (preferably broad-spectrum antibiotics)
- **Adults**: IV ceftriaxone 2g once daily 5 days.
- For patients who are critical, hospitalized, immunocompromised or have previous structural lung disorder: Ceftazidime/Cefepime 2g IV three times per day (TID) +/- Vancomycin 1g IV BID or + Azithromycin 500mg PO daily for 3 days
- Meropenem 1g IV every 8hours +/- Vancomycin 1g IV every12 hours in critical patients if there is no response with the above alternative or culture and sensitivity result is suggestive.
- IV Metronidazole 500mg three times per day can be added when aspiration pneumonia is suspected (usually 7 days).
- When patients improve and are able to take PO, Amoxicillin-Clavulanate (Augmentin) 2g PO BID for 7-10 days
- **Children**: IV ceftriaxone 50-100mg/kg daily in divided doses (usually for 7 days)

**Anti-pyretic and analgesics:**

**Adults:**

- Paracetamol 1g PO every 6–8 hours. Maximum 4g/ 24hr
- Tramadol 50–100mg PO/IV every 4–6 hours as needed, daily maximum 400 mg/day can be given alternatively or combined with Paracetamol.

**Children:**

- Paracetamol10–15mg/kg every 6 hourly, maximum dose 60mg/kg/day
- Children > 6 months for analgesics purpose tramadol 1–2mg/kg every 4–6 hours, maximum 400mg/day can be given alternatively or combined with Paracetamol.

1. **Management of Acute Hypoxemic Respiratory Failure secondary to ARDS**

- Oxygen via face mask with reservoir bag-flow rates 10-15L/min
- High-flow nasal oxygen/non-invasive ventilation should only be used in selected patients without comorbidities and for non-pregnant patients.
- Monitor closely for one hour and deliver invasive ventilation if patients acutely deteriorate or have no improvement.
- A trained and experienced provider using airborne precautions should perform endotracheal intubation.
- MV setting-low tidal volume (4-8ml /kg), low inspiratory pressure, high positive end-expiratory pressure (PEEP)
- If no improvement, consider prone ventilation.

1. **Management of Septic Shock**

Apply the Six Sepsis Management Bundles with in 1hr: appropriate fluid management, Oxygen delivery, antibiotics, sending specimen for culture and sensitivity, and monitoring of lactate and urine out-put hourly.

- Immediate aggressive volume expansion with isotonic solution, preferably R/L or alternatively with N/S, is the main stay of treatment during septic shock.
- **Adults**: start with at least 30ml/kg in the first 3hrs, then additional fluid boluses.
- **Children**: 20ml/kg as rapid bolus and up to 40-60ml/kg in the first 1hr.
- Further fluid administration depends on the response to the previous fluid resuscitations.
- Closely monitor for signs of fluid overload (jugular venous distension, crackles on lung auscultation, pulmonary edema on imaging, or hepatomegaly in children)
- Stop or decrease fluid administration if signs of fluid overload are identified.
- Watch also for signs of target perfusion achievement (Mean Arterial Pressure (MAP)>65 mmHg or age appropriate target for children, urine output (>0.5 ml/kg/hr in adults, 1 ml/kg/hr in children), and improvement of skin mottling, capillary refill, level of consciousness)
- If target perfusion is not achieved or hemodynamic response is poor with standard fluid administration within one hour, start vasopressor administration.
- The vasopressor of choice in adults is norepinephrine (NE) (2-30 μg/min/ (0.1-1 μg/kg/min) but epinephrine (2-30 μg/min, (0.1-1 μg/kg/min) and dopamine (2-20 μg/kg/min) can be used respectively. Titrate dose based on response.
- For children, epinephrine (0.1–0.3 μg /kg/min) is the first-line vasopressor.
- Closely monitor the veins for any extravasations of vasopressors as it may cause tissue swelling and necrosis.
- Broad-spectrum antibiotics should be administered for possible superimposed infection.
- **Adults**:
- In patients who are critical, hospitalized, immunocompromised or have previous structural lung disorder: Ceftazidime/Cefepime 2g IV TID +/-Vancomycin 1g IV BID
- Meropenem 1g IV every 8hours +/- Vancomycin 1g IV every 12 hours in critical patients if there is no response with the above alternative or culture and sensitivity result is suggestive
- When patients improve and are able to take PO, Amoxicillin -clavulanate (Augmentin) 2g PO BID for 7-10 days
- **Children**: IV ceftriaxone 80mg/kg daily in divided doses (usually for 7-10 days)
- Other antibiotics can be administered based on the clinical judgment of the clinician
- Surgical drainage or debridement of an abscess or dead /necrotized tissue
- Blood transfusion if hemoglobin is ≤ 7mg/dl to keep adequate O2 saturation
- Collect complete blood count, organ function tests, electrolytes, and imaging results and act accordingly.

**References**

1. World Health Organization. 2020. Clinical management of COVID-19: Interim guidance. Geneva, Switzerland, May.
2. Ethiopian Public Health Institute and Ethiopia Ministry of Health. 2020. Case management protocol for corona virus disease-19 (COVID-19) in Ethiopia. Addis Ababa, Ethiopia, March.
