# Supplementary material for: Hospitalization costs for COVID-19 in Ethiopia: Empirical data and analysis from Addis Ababa’s largest dedicated treatment center
Source: PLoS One. 2022 Jan 21;17(1):e0260930. doi: 10.1371/journal.pone.0260930 (PMC8782501; doi:10.1371/journal.pone.0260930)
Supplement: S2 Appendix — (DOCX) [file pone.0260930.s002.docx]

**Supplementary web appendix II**

1. **Cost computation for hospitalization costs for COVID-19 in Ethiopia**

Cost is computed from the provider’s perspective and only direct medical costs borne by the provider were included. To compute costs, we used ingredients-based approach, which calculated costs by multiplying quantities of inputs by their unit prices.

In the following section, we present the details of cost computations for the different cost categories.

**Cost inputs**

- Total number of cases (A) = 2,543
- Number of patients in critical condition (B) = 235
- Number of patients with severe disease (C) = 515
- Number of patients with moderate disease (D) = 1,841
- The mean patient length of stay for critical cases (E) = 19.2 days
- The mean patient length of stay for severe disease (F) = 11.3 days
- The mean patient length of stay for moderate disease (G) = 9.2 days
- Total bed days for critical cases (H) = B*E
- Total bed days for severe cases (I) = C*F
- Total bed days for moderate cases (J) = D*G
- Total bed days for all patients in the study period (K) = H + I + J
- Proportion of bed days from the total bed days for case with critical disease (L) is computed as:

$$L= \frac{H}{K}$$

- Proportion of bed days from the total bed days for case with severe disease (M) is computed as:

$$M= \frac{I}{K}$$

- Proportion of bed days from the total bed days for case with moderate disease (N) is computed as:

$$N= \frac{J}{K}$$

**Cost computation**

1. **Drugs and supplies cost**

- We listed and quantified all the drugs that were prescribed to patients over the study period (March to November, 2020) by level of disease severity
- For each drug we calculated the total amount consumed by level of disease severity and the cost is computed by multiplying total amount consumed by the unit price
- The total drugs and supplies cost is the sum of drugs and supplies cost for patients with critical disease plus drugs and supplies cost for severe disease plus drugs and supplies cost for moderate disease
- Drugs and supplies cost per treated episode were computed as follows:
- For patients with critical disease:

$$=\frac{Total drugs and supplies cost for critical disease}{B}$$

- For patients with severe disease:

$$=\frac{Total drugs and supplies cost for severe disease}{C}$$

- For patients with moderate disease:

$$=\frac{Total drugs and supplies cost for moderate disease}{D}$$

- Drugs and supplies cost per bed day were computed as follows:
- For patients with critical disease:

$$=\frac{Total drugs and supplies cost for critical disease}{H}$$

- For patients with severe disease:

$$=\frac{Total drugs and supplies cost for severe disease}{I}$$

- For patients with moderate disease:

$$=\frac{Total drugs and supplies cost for moderate disease}{J}$$

1. **Laboratory and diagnostic test cost**

- We identified and listed all the laboratory and diagnostic tests that were made to patients over the study period (March to November, 2020)
- For each laboratory test or diagnostic test we calculated the total amount consumed by level of disease severity and the cost is computed by multiplying total amount consumed by the unit price of the test
- The total laboratory and diagnostic cost is the sum of all laboratory and diagnostic tests cost.
- Laboratory and diagnostic test cost per treated episode were computed as follows:
- For patients with critical disease:

$$=\frac{Total laboratory and diagnostic test cost for critical disease}{B}$$

- For patients with severe disease:

$$=\frac{Total laboratory and diagnostic test cost for severe disease}{C}$$

- For patients with moderate disease:

$$=\frac{Total laboratory and diagnostic test cost for moderate disease}{D}$$

- Drugs and supplies cost per bed day were computed as follows:
- For patients with critical disease:

$$=\frac{Total laboratory and diagnostic test cost for critical disease}{H}$$

- For patients with severe disease:

$$=\frac{Total laboratory and diagnostic test cost for severe disease}{I}$$

- For patients with moderate disease:

$$=\frac{Total laboratory and diagnostic test cost for moderate disease}{J}$$

1. **Personnel cost**

- For each staff including support staff, we collected data on gross monthly salary, allowances and duty time payments over the study period
- Total personnel cost is computed as the sum of gross monthly salary, allowances and duty time payments for all the staff at the center
- We then calculated the share of personnel cost for each disease severity using proportion of bed days as follows:
- Total personnel costs for critical disease (O) =$\left( Total personnel cost \right)*L$
- Total personnel costs for severe disease (P) =$\left( Total personnel cost \right)*M$
- Total personnel costs for moderate disease (Q) =$\left( Total personnel cost \right)*N$
- Personnel cost per treated episode were computed as follows:
- For patients with critical disease = $\frac{O}{B}$
- For patients with severe disease = $\frac{P}{C}$
- For patients with moderate disease = $\frac{Q}{D}$
- Personnel cost per bed day were computed as follows:
- For patients with critical disease = $\frac{O}{H}$
- For patients with severe disease = $\frac{P}{I}$
- For patients with moderate disease = $\frac{Q}{J}$

1. **Meal cost**

- Meals were served three times per day for all the staff and patients at the center over the study period. For each month, we identified the total number of meals received and we summed up to get total number of meals received over the study period.
- We calculated the total meal cost (R) as follows: total number of meals received multiplied by the price per meal.
- We then calculated the share of meal cost for each disease severity using proportion of bed days as follows:
- Total meal cost for critical disease (S) =$R*L$
- Total meal cost for severe disease (T) =$R*M$
- Total meal cost for moderate disease (U) =$R*N$
- Meal cost per treated episode were computed as follows:
- For patients with critical disease = $\frac{S}{B}$
- For patients with severe disease = $\frac{T}{C}$
- For patients with moderate disease = $\frac{U}{D}$
- Meal cost per bed day were computed as follows:
- For patients with critical disease = $\frac{S}{H}$
- For patients with severe disease = $\frac{T}{I}$
- For patients with moderate disease = $\frac{U}{J}$

1. **Building cost**

- We measured each room to get the total surface area in square meter (m^2^) of buildings at the center.
- We got an estimate of rental prices per m^2^ from buildings in the neighborhood (we took the average of several buildings)
- Total building cost (V) = (total surface area)*(rental price per m^2^)
- We then calculated the share of building cost for each disease severity using proportion of bed days as follows:
- Total building cost for critical disease (W) =$V*L$
- Total building cost for severe disease (X) =$V*M$
- Total building cost for moderate disease (Y) =$V*N$
- Building cost per treated episode were computed as follows:
- For patients with critical disease = $\frac{W}{B}$
- For patients with severe disease = $\frac{X}{C}$
- For patients with moderate disease = $\frac{Y}{D}$
- Building cost per bed day were computed as follows:
- For patients with critical disease = $\frac{W}{H}$
- For patients with severe disease = $\frac{X}{I}$
- For patients with moderate disease = $\frac{Y}{J}$

1. **Vehicle cost**

- We collected data on the number and types of vehicles used by the center. We also collected rental price information by car type and capacity
- Total vehicle cost (Z) over the study period was calculated by multiplying number of cars by rental prices.
- We then calculated the share of vehicle cost for each disease severity using proportion of bed days as follows:
- Total vehicle cost for critical disease (AA) =$Z*L$
- Total vehicle cost for severe disease (BB) =$Z*M$
- Total vehicle cost for moderate disease (CC) =$Z*N$
- Vehicle cost per treated episode were computed as follows:
- For patients with critical disease = $\frac{AA}{B}$
- For patients with severe disease = $\frac{BB}{C}$
- For patients with moderate disease = $\frac{CC}{D}$
- Vehicle cost per bed day were computed as follows:
- For patients with critical disease = $\frac{AA}{H}$
- For patients with severe disease = $\frac{BB}{I}$
- For patients with moderate disease = $\frac{CC}{J}$

1. **Utilities cost**

- We collected expenditures for fuel (vehicles use), electricity and water
- Total utilities cost (DD) was the sum of expenses for fuel, electricity and water over the period
- We then calculated the share of utilities cost for each disease severity using proportion of bed days as follows:
- Total utilities cost for critical disease (EE) =$DD*L$
- Total utilities cost for severe disease (FF) =$DD*M$
- Total utilities cost for moderate disease (GG) =$DD*N$
- Utilities cost per treated episode were computed as follows:
- For patients with critical disease = $\frac{EE}{B}$
- For patients with severe disease = $\frac{FF}{C}$
- For patients with moderate disease = $\frac{GG}{D}$
- Utilities cost per bed day were computed as follows:
- For patients with critical disease = $\frac{EE}{H}$
- For patients with severe disease = $\frac{FF}{I}$
- For patients with moderate disease = $\frac{GG}{J}$

1. **Equipments**

- We identified and listed all the medical equipments and beds used at the center
- For each equipment, we estimated equivalent annual cost (EAC) using the following formula:

$EAC= \frac{Asset price*Discount rate}{\begin{matrix} 1 - & {(1+Discount rate)}^{-n} \end{matrix}}$ [1,2]

, where n is the expected service life year [3].

- We then adjusted the EAC for the study period (256 days)
- Total equipment cost (HH) is the sum of all equipments cost
- We then calculated the share of equipments cost for each disease severity using proportion of bed days as follows:
- Total equipments cost for critical disease (II) =$HH*L$
- Total quipments cost for severe disease (JJ) =$HH*M$
- Total quipments cost for moderate disease (KK) =$HH*N$
- Equipments cost per treated episode were computed as follows:
- For patients with critical disease = $\frac{II}{B}$
- For patients with severe disease = $\frac{JJ}{C}$
- For patients with moderate disease = $\frac{KK}{D}$
- Equipments cost per bed day were computed as follows:
- For patients with critical disease = $\frac{II}{H}$
- For patients with severe disease = $\frac{JJ}{I}$
- For patients with moderate disease = $\frac{KK}{J}$

**Total cost =** **Drugs and supplies cost + Laboratory and diagnostic costs + Personnel costs + Meal costs + Building cost + Vehicles cost + Utilities cost + Equipment cost**

**References**

1. Drummond MF, Sculpher MJ, Torrance GW, O'Brien BJ, Stoddart GL. Methods for the Economic Evaluation of Health Care Programmes. Third Edition ed: OXFORD UNIVERSITY PRESS; 2005.
2. American Hospital Association. 1998. Estimated Useful Lives of Depreciable Hospital Assets. 29/01/2021. Available at: <https://www.google.com/url?sa=t&rct=j&q=&esrc=s&source=web&cd=&cad=rja&uact=8&ved=2ahUKEwj64L->
3. Tan-Torres Edejer T, Baltussen R, Adam T et al. 2003. WHO Guide to Cost-Effectiveness Analysis. Geneva: World Health Organization.
4. **Results: supplementary tables**

Table 1. Number of monthly RT-PCR positive COVID-19 patients admitted to Ekka Kotebe treatment center and mean hospital stay (days) by level of disease severity.

|  | Moderate | | Severe | | Critical | |
| --- | --- | --- | --- | --- | --- | --- |
|  | Number of patients (n) | Mean hospital stay  (days) | Number of patients (n) | Mean hospital stay (days) | Number of patients (n) | Mean hospital stay (days) |
| March | 23 | 10 | 18 | 11 | 8 | 21 |
| April | 74 | 5 | 14 | 9 | 2 | 20 |
| May | 515 | 12 | 36 | 10 | 28 | 22 |
| June | 314 | 6 | 15 | 14 | 15 | 19 |
| July | 471 | 11 | 89 | 11 | 43 | 19 |
| August | 339 | 7 | 116 | 13 | 48 | 16 |
| September | 48 | 8 | 76 | 10 | 37 | 18 |
| October | 17 | 9 | 83 | 11 | 26 | 23 |
| November | 40 | 6 | 68 | 11 | 28 | 20 |
| Total | 1,841 | 9 | 515 | 11 | 235 | 19 |
| Moderate = COVID-19 patients with pneumonia  Severe = COVID-19 patients with severe pneumonia  Critical = COVID-19 patients with any of the following: acute respiratory distress syndrome, sepsis, or septic shock | | | | | | |

Table 2. Total and monthly costs by different cost category in 2020 USD at Ekka Kotebe COVID-19 treatment center.

|  | Food | Personnel | Drugs & supplies | Capital^^^ | Laboratory &  diagnostics | Other^±^ | Total |
| --- | --- | --- | --- | --- | --- | --- | --- |
| March | $36,360 | $313,535 | $7,747 | $19,994 | $1,264 | $1,003 | $379,903 |
| April | $87,850 | $314,576 | $14,545 | $37,489 | $2,373 | $1,862 | $458,696 |
| May | $297,395 | $197,254 | $83,636 | $38,739 | $13,642 | $1,924 | $632,590 |
| June | $203,154 | $93,979 | $54,387 | $37,489 | $8,871 | $1,862 | $399,742 |
| July | $337,583 | $94,437 | $95,336 | $38,739 | $15,550 | $1,924 | $583,568 |
| August | $290,302 | $94,437 | $79,526 | $38,739 | $12,972 | $1,924 | $517,899 |
| September | $125,827 | $94,362 | $25,455 | $37,489 | $4,152 | $1,862 | $289,147 |
| October | $83,214 | $94,437 | $19,921 | $38,739 | $3,249 | $1,924 | $241,483 |
| November | $85,105 | $94,362 | $21,502 | $37,489 | $3,507 | $1,862 | $243,828 |
| Total | $1,546,790 | $1,391,379 | $402,055 | $324,905 | $65,580 | $16,147 | $3,746,855 |
| Capital costs include cost of buildings, cost of equipments and vehicles.  ^±^Other costs include cost of fuel for vehicles and utilities. | | | | | | | |
